# *In vitro* and *in vivo* efficacy of the Active Oligo Skin complex™, a new active ingredient processed from seawater, on multiple parameters of atopic skin

**DOI:** 10.1093/skinhd/vzae029

**Published:** 2025-02-14

**Authors:** Nicolas Lebonvallet, Chloé Catovic, Marc Feuilloley, Raphael Leschiera, Alexia Reux, Matthieu Talagas, Ianis Cousin, Laurent Misery, Emilie Simon, Sylvie Chopin, Johan Gardères

**Affiliations:** University of Brest, LIEN, Brest, France; Unité de Recherche 4312 Communication Bactérienne et Stratégies Anti-infectieuses (UR CBSA), Université de Rouen Normandie, Evreux, France; Unité de Recherche 4312 Communication Bactérienne et Stratégies Anti-infectieuses (UR CBSA), Université de Rouen Normandie, Evreux, France; Centre d'Expertise Cosmetomics@URN, Université de Rouen Normandie, Evreux, France; University of Brest, LIEN, Brest, France; University of Brest, LIEN, Brest, France; University of Brest, LIEN, Brest, France; University of Brest, LIEN, Brest, France; University of Brest, LIEN, Brest, France; Laboratoires Gilbert, R&D Department, Hérouville St Clair, France; Laboratoires Gilbert, R&D Department, Hérouville St Clair, France; Laboratoires Gilbert, R&D Department, Hérouville St Clair, France

## Abstract

**Background:**

Different symptoms are associated with atopic skin, including dryness, pruritus and pain, and affect patients’ quality of life. The environment, microbiota, epidermis, immune and nerve cells are all implicated in the pathogenesis of atopic skin. *Staphylococcus aureus* is the focus of particular attention. Epidermis is implicated at multiple levels: inflammatory process, barrier, control of moisture and water loss. Sensory neurons that participate in cutaneous neurogenic inflammation and pruritus are seen as a potential new target. Specific management strategies and new treatments for adults and children are needed to help in more refractory cases. As a baseline of management, guidelines recommend a treatment to moisturize the skin and maintain the skin barrier function, such as an emollient.

**Objectives:**

To evaluate a new product *in vitro* and *in vivo* in order to validate the potential of its use in people with atopic skin or dry skin.

**Methods:**

A specific mineral composition, Active Oligo Skin complex™, from seawater was developed and included in a balm. The effects of a solution and balm containing the complex were evaluated *in vitro* on the growth and biofilm formation of *Staphylococcus aureus* and *Staphylococcus epidermidis* in different skin models, and *in vivo* in adult and young volunteers.

**Results:**

*In vitro*, the complex modulated bacterial biofilm formation and growth, decreased cytokine [interleukin (IL)-1, IL-6, IL-4] and neuropeptide (substance P) release, and increased the expression of CL1 and CL4. On volunteers with dry skin, the complex had a moisturizing effect after 1 h of application. Dryness and roughness were also reduced in young participants with atopic skin. The balm decreased erythema and pruritus after 21 days of topical application on 60 young participants. On 22 adult participants, stinging score was decreased after ­application.

**Conclusions:**

The Active Oligo Skin complex™ appears to display potent antipruritic and anti-inflammatory activities, both *in vitro* and *in vivo*.

## Teaser

In our paper, the Active Oligo Skin complex™, processed from sea water and possessing specific mineral composition, was developed and included in a balm. The effects of solution and balm containing the complex were evaluated *in vitro* on bacteria growth and biofilm formation of *S. aureus* and *S. epidermidis*, on different skin models, and *in vivo* on adult and young volunteers. In summary, the Active Oligo Skin complex™ appears to display *in vitro* and *in vivo* potent antipruritic and anti-inflammatory activities.

**What is already known about this topic?**

- New treatments with specific management strategies for adults and children with atopic skin are needed to help more refractory patients.

**What does this study add?**

- Atopic Active Oligo Skin complex™ appears to have *in vivo* antipruritic activity and a moisturizing effect.

Atopic dermatitis (AD) is a chronic and pruritic inflammatory skin disorder with a complex pathogenesis, the diagnosis of which is based on clinical aspects and patient history.^1^ Different symptoms are associated with AD, including dryness, pruritus and pain, affecting patients’ quality of life (QoL). Cellular and tissue mechanisms involved in AD include a complex interplay between different actors: the environment, microbiota, epidermis, and immune system and nerve cells. *Staphylococcus aureus* is particularly implicated, and is the focus of attention for potent direct or indirect therapies.^2^ Epidermis is implicated at multiple levels: in the inflammatory process, as a barrier, and in controlling moisture and water loss. Dysregulation between the different actors leads to engagement in inflammatory self-maintenance, neurogenic inflammation, pruritus and alteration of the epidermal barrier.^3–10^ Inflammatory interactions are mediated by interleukins (IL)-4, IL-13 and IL-31 produced by immune cells; thymic stromal lymphopoietin, IL-25 and IL-33 by keratinocytes; and neuropeptides like brain natriuretic peptides, released by sensory neurons, keratinocytes and endothelial cells.^7,11,12^ Different junction proteins such as Zonula Occludens 1 (ZO1), Claudin-1 (CL1) or Claudin-4 (CL4) are implicated in the barrier and their expression is dysregulated in lesional or nonlesional skin in patients with AD.^10,13–16^ Specific management strategies and new treatments for adults and children are needed.^1,12,17–20^ As the baseline of AD management, guidelines recommend a treatment to moisturize the skin and maintain skin barrier function, such as an emollient or oil baths with specific characteristics and applications (free of fragrance or other contact allergens).^1^ For more severe AD, glucocorticosteroids are proposed, but are not recommended for chronic use in order to avoid side-effects, such as skin atrophy. For prevention or during crisis phases, anti-inflammatory agents coupled with emollient balms can be used, according to the severity of AD. Minerals are also envisaged as a potential tool to reduce AD severity. Inorganic elements are implicated in skin ­homeostasis.

Complementary to the positive effects of mineral-­enriched baths on skin disease, anti-inflammatory, immunomodulatory, skin barrier modulatory and/or antibacterial effects have been demonstrated with several mineral components of spring waters and refined seawater, because of the presence of H_2_S, zinc, magnesium, manganese, copper, sodium or calcium, or specific combinations of these ions.^21–29^ In addition to the mineral quality of substances and the final formulation, another concern around the microbiological cosmetic safety of these agents is their potential impact on the cutaneous microbiota. This microbiota is not only essential for protection against environmental pathogens and physical stress, but also plays a key role in training the immune system and contributes to lesion repair.^30–35^

In this study, we assessed an active mineral solution, the Active Oligo Skin (AOS) complex™, processed from ­seawater, *in vitro* and *in vivo* with a double approach in the forms an emollient balm and a unique ionic formula, presenting potent anti-inflammatory, microbiota modulatory and skin barrier-restructuring effects. Firstly, we analysed the effect of AOS complex on normal and AD skin bacteria. Secondly, we evaluated the balm and AOS complex efficacy in different *in vitro* inflammatory models. Finally, the AOS complex™ was assessed on pruritus, unpleasant sensation, dryness and moisture, on volunteers with atopic skin.

## Materials and methods

The AOS complex™ is an isotonic solution obtained from seawater, drawn from the Iroise marine UNESCO park and using the procedure according to the patents FR 2 872 046/FR 1 853 214. Briefly, the seawater is first rid of its impurities and microorganisms by ultrafiltration. It then undergoes electrodialysis to deplete it of monovalent ions (selective membranes). The remaining trace elements in the retentate are concentrated by reverse osmosis nanofiltration to form the solute. The emollient balm is composed of 50% of the AOS complex and shea butter, glycerin, virgin coconut oil, allantoin, pentavitin and alpha-glucan oligosaccharide. The placebo balm is composed of the same ingredients except for the AOS complex, which is substituted by purified water. For the *in vitro* and *in vivo* tests, AOS complex was also used to evaluate in solution at 100% and at 50%, or for biofilm studies at 10%, diluted with purified water. The supplementary method is provided as Supporting Information.

### Effect of the AOS complex on planktonic bacterial growth and biofilm formation

Bacterial strains and culture conditions are detailed in the Supporting Information. *Staphylococcus epidermidis* and *S. aureus* were studied in the presence of AOS complex or physiological water NaCl 0.9% (PW). Growth kinetics at 37 °C were determined by measuring optical density at 580 nm (OD_580nm_). Biofilm formation was studied in 24-well flat-bottomed polystyrene plates following a procedure adapted from O’Toole^36^ by measuring crystal violet staining at OD_595nm_. Results were expressed as a percentage of the control nontreated bacteria values. Statistical tests were performed with Past 3.x software and the Student’s *t*-test. Biofilm formation was equally analysed by confocal laser scanning microscopy. *Staphylococcus epidermidis* was stained with Syto 9 Green Fluorescent Nucleic Acid. The *S. aureus* strain contained mCherry. Syto 9 and mCherry fluorescence emissions were detected at 498 and 610 nm, respectively. For visualization and processing of three-­dimension images, Zen 2.1 SP1 software was used. Quantitative analyses of image stacks were performed with COMSTAT2 software. Biomass volume (μm^3^/μm^2^), and maximal and average thickness (μm) were determined using ImageJ software.

### Effect of the AOS complex on inflammatory *in vitro* models

For ZO1, CL1 and CL4 evaluation, human skin explants were incubated at an air–liquid interface for 7 days in Dulbecco’s modified Eagle’s medium (DMEM)/F12. At 5 days, the AOS complex (50% or 100%) mixed in 4% methyl cellulose gel, placebo (gel not supplemented with AOS complex, but replaced with PW), balm and placebo balm were applied. After RNA extraction, gene expression modulation was evaluated using the 2^−ΔΔct^ method, with actin as the housekeeping gene. Probes designed for the study are presented in Table S1 (see Supporting Information).

For IL-4 evaluation, dorsal root ganglia cells (DRG)^37–39^ and skin explant were prepared as previously described to constitute a reinnervated skin model.^40,41^ Balm, placebo or the gel with or without AOS complex were deposited at the surface of skin explants. Five minutes later, lactic acid (LA; 10% in phosphate-buffered saline) was applied. After 24 h of culture, supernatants were collected and IL-4 was quantified using a Duoset kit according to the manufacturer’s instructions (R&D Systems, Minneapolis, MN, USA).

Regarding IL-6, IL-1 and tumour necrosis factor (TNF)-α, skin explants were placed in DMEM for 4 h and subsequently pretreated for 24 h with gels containing the AOS complex at 0%, 2%, 10% or 50%, or dexamethasone as control. Then, phorbol 12-myristate 13-acetate (PMA) was applied to skin explants previously treated or not by the formula. After 24 h of incubation at 37 °C, supernatants were collected to quantify IL-6, IL-1 and TNF-α with an enzyme-linked immunosorbent assay kit (Magnetic Luminex Performance Assay, Human Cytokine Premixed Kit A, FCSTM03-03, panel jxH3AMLL; R&D Systems, Minneapolis, MN, USA).

Regarding substance P (SP), after 3 days of culture of DRG cells, the medium was removed, and human primary keratinocytes were added in culture for an additional day in KSFM^®^ (Gibco, Paisley, UK). For assessment of SP release (#583751; Cayman Chemical, Ann Arbor, MI, USA) capsaicin [transient receptor potential cation channel subfamily V member 1 (TRPV1) agonist] 10 µmol L–1 alone dissolved in dimethysulfoxide (DMSO 0.02%), medium with capsaicin and AOS complex at 50% or 100%, or capsaicin solvent (DMSO 0.02%) alone were used.

### *In vivo* evaluation of the effect of the AOS complex on skin moisturizing and stinging sensation in adults, and on atopic skin symptoms and quality of life in young patients

The moisturizing potential of the AOS complex was determined by repeated measurements of the skin’s electrical capacity (Corneometer^®^; Courage + Khazaka electronic, Köln, Germany) on treated (T) sites and nontreated (NT) control sites. Measurements were realized with AOS complex in balm form on 11 women volunteers (average age 61 years) with dry leg skin, and at 50% and 100% on 10 other volunteers. Stinging sensation was evaluated with a classic methodology^42–44^ using a randomized site on 22 women volunteers (average age 44 years) for the product containing 50% AOS complex and on 22 women volunteers (average age 38 years) for the product with 100% AOS complex. T and NT sites were analysed. Quality of life, skin aspect (dryness, erythema, roughness) and sensation of pruritus were evaluated in 33 participants aged 6 months to 3 years, and in 33 participants aged 3 to 17 years with a pruritus score ≥3 on a scale from 0 to 10 and with AD (not in crisis) (symptoms based on parents’ declarations). The product was applied each day for 21 days (D21) and the study was completed by two after 2 more days without application of the product (D23).

### Statistics

Normality was evaluated with a Shapiro–Wilk test. If the normality test was passed, a parametric test was employed using a *t*-test for two comparisons or a one-way ANOVA with Dunnett’s post hoc test for multiple comparisons (adapted to paired data if data were paired). If the normality test was not passed, a nonparametric test was used, either the Mann–Whitney or the Wilcoxon test if appaired data. *N* was used for independent experiments or volunteers, *n* for technical replicates.

## Results

### AOS complex modifies the microbiota biofilm formation

**Figure 1:**
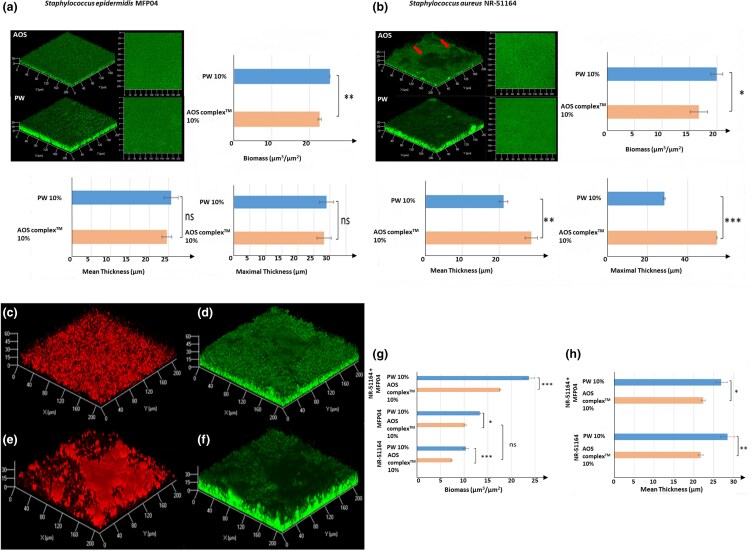
Mature biofilm formed after 24 h by (a) *Staphylococcus epidermidis* MFP04 and (b) *Staphylococcus aureus* NR-51164 grown in pure cultures in the presence of the Active Oligo Skin (AOS) complex 10% or physiological water 10% (PW). Three-dimensional views of the biofilms and top views were generated by confocal laser microscopy and analysed by COMSTAT2 (www.comstat.dk) for calculation of the respective biomass, mean thickness and maximal thickness. Arrows indicate ‘mushroom-like’ structures that were particularly abundant in *S. aureus* biofilms exposed to the AOS complex. These structures were associated with an increase of the maximal biofilm thickness. *P < 0.05; **P < 0.01; ***P < 0.001; *N* = 4. Mature biofilm formed after 24 h by *S. aureus* NR-51164 and *S. epidermidis* MFP04 grown in mixed cultures in the presence of the AOS complex 10% or physiological water 10% (C-H). The mCherry-labelled *S. aureus* NR-51164 strain grown with (c) AOS complex or (e) PW was visualized in red at 543 nm. Biofilms formed by total bacteria (i.e. *S. aureus* and *S. epidermidis*) exposed to (d) AOS complex or (f) PW were visualized in green at 488 nm after labelling with Syto9. COMSTAT2 was used for calculation of the biofilms (g) biomass and (h) mean thickness. The biomass formed by *S. epidermidis* MFP04 was calculated by subtraction of the *S. aureus* biomass in the total mixed biofilm biomass. *P < 0.05; **P < 0.01; ***P < 0.001; *N* > 3.ns, non significant (*P* > 0.05).

To evaluate the effect of the AOS complex™ on the micro­biota, as a first step, we investigated its impact on the growth kinetics of *S. aureus* and *S. epidermidis* present on healthy and AD skin.^45^ In liquid medium, the complex (10%) had no effect on *S. epidermidis* or *S. aureus* growth (data not shown). On mature biofilms, a limited decrease in *S. epidermidis* biomass (−11%) (Figure 1a) and no effect on biofilm formation was induced by the complex. The *S. aureus* biofilm biomass was reduced (−15%), but the mean and maximal thickness were increased (+45%) with the formation of ‘mushroom-like’ defence structures (Figure 1b, red arrows). When both bacteria formed binary biofilms, as shown on Figure 1, biomass was reduced because of *S. epidermidis* adhesion reduction (Figure 1g). The complex (10%) had a higher inhibitory effect on *S. aureus* (−28%) than on *S. epidermidis* (−20%) final biomass, and no *S. aureus* ‘mushroom-like’ defence structures were visualized (Figure 1g). The complex induced a reduction in biofilm formation (Figure 1h).

### AOS complex partially reduces cytokine release in different inflammation models and increases expression of junction proteins

**Figure 2:**
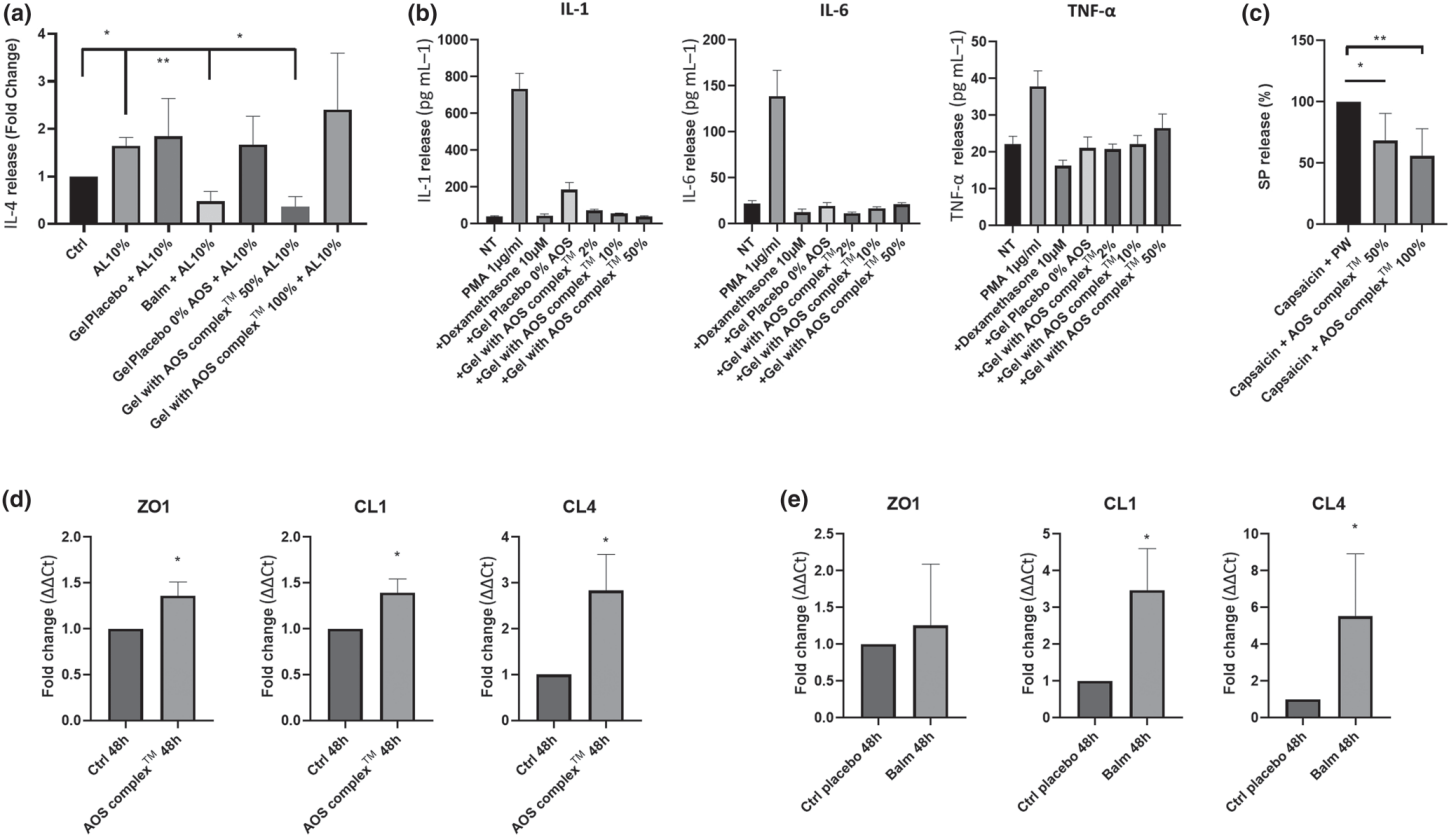
*In vitro* inflammation model. Active Oligo Skin (AOS) complex was evaluated in different inflammatory models. (a) After 9 days of culture, reinnervated skin explant was exposed to lactic acid (LA) at 10% and complex in balm, in 4% methyl cellulose gel or respective control (PW NaCl gel or balm without complex). Graph represents mean (SEM) of interleukin (IL)-4 fold-change release compared with the control normalized at 1 for each independent experiment (Kruskal–Wallis post-hoc Dunn, *P < 0.05, **P < 0.01; *N* > 4). (b) Noninnervated skin explants were treated, before the addition of PMA, for 24 h with complex at 0%, 2%, 10% or 50%, or control without formula. Graphs represent the mean (SEM) of IL-1, IL-6 and tumour necrosis factor (TNF)-α release of three independent skin explants from one donor (*n* = 3). (c) Substance P (SP) release evaluation in a co-culture between dorsal root ganglia cells, including sensory neurones and keratinocytes, after the addition of capsaicin at 10 µg mL–1 for 15 min with or without complex at 50% or 100%. Graphs represent mean (SEM) of the percentage of SP release compared with control induction with capsaicin alone at 100% in independent experiments experiment (Kruskal–Wallis post-hoc Dunn, *P < 0.05; **P < 0.01; *N* = 6). Analysis of effect of AOS complex on junction expression on skin explant. Non-reinnervated skin explants were pretreated with complex in (d) 4% methyl cellulose gel or (e) in balm with their respective control 2 days before the end of culture for 7 days. Graphs represent mean (SEM) fold change obtained by the quantitative polymerase chain reaction ΔΔct method for independent experiments (Mann–Whitney, one-sided, *P < 0.05; *N* = 3). CL1, Claudin-1; CL4, Claudin-4; Ctrl, control; NT, nontreated; PMA, phorbol 12-myristate 13-acetate; PW, physiological water; ZO1, Zonula Occludens-1.

To determine the mediators modulated by the AOS complex, different *in vitro* inflammation models were employed. A first model used re-innervated skin explants exposed to LA 10% as used in the stinging test. Results showed that the AOS complex in balm or formulated at 50% in gel significantly decreased IL-4 release (−72% and −74%, respectively) in supernatant induced by LA. Placebo balm and 100% ­complex-containing gel did not show a significant reduction in IL-4-induced release (Figure 2a). On skin explant induced by PMA, the complex at 2%, 10% and 50% induced a decrease in IL-1 release. AOS complex at 2% led to a decrease in IL-6 release but not when used at 10% and 50% (Figure 2b). The complex did not modify TNF-α release induced by PMA for all tested concentrations. The anti-inflammatory positive control dexamethasone induced a decrease in IL-1, IL-6 and TNF-α release. In a co-culture model of neurones and keratinocytes with inflammation induced by capsaicin (100% of SP release), the complex used at 50% and 100% induced a ­significant decrease of SP release (−46% and −48%, respectively) (Figure 2c).

Intercellular junctions are associated with regulation of the barrier function of the epidermis. In order to correlate the effects of the complex with skin barrier function and permeability, we used it on models inducing alteration of junction protein expression. In skin explant in culture, the complex formulated in gel after 48 h induced a mean (SEM) increase of CL1, CL4 and ZO1 by 1.39 (0.15), 2.8 (0.79) and 1.36 (0.15), respectively (Figure 2d), and of CL1 and CL4 when formulated in balm by 3.5 (1.1) and 5.5 (3.4), respectively (Figure 2e).

### AOS complex reduces stinging sensation and atopic symptoms, and increases skin moisture and quality of life in patients with atopic dermatitis

**Figure 3:**
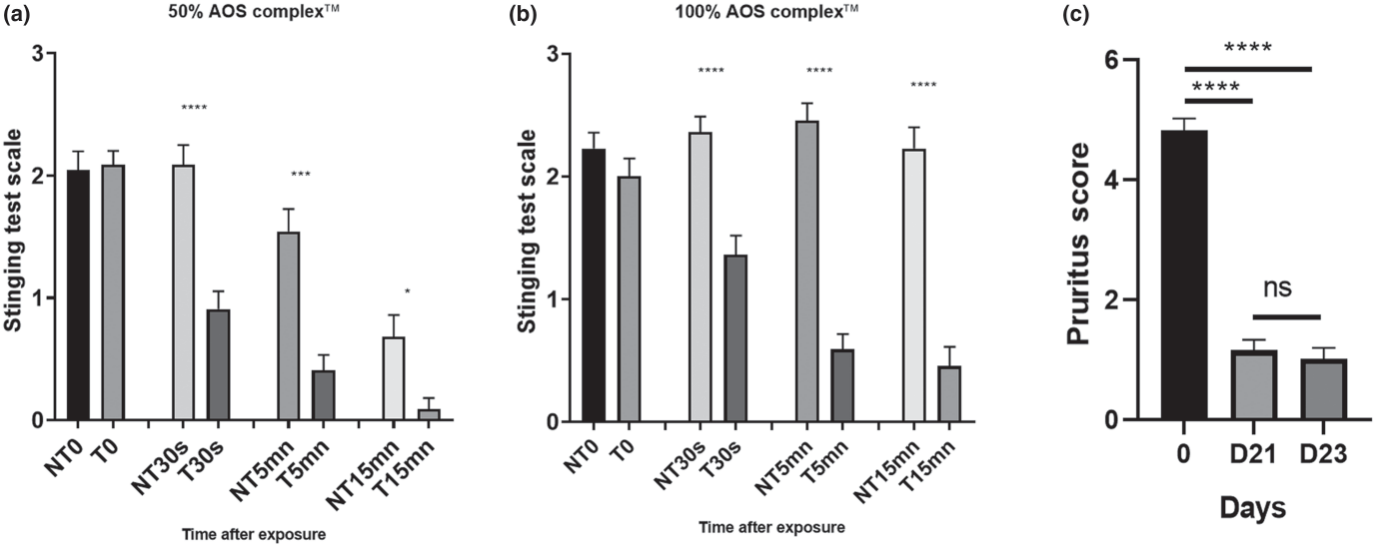
Effect of Active Oligo Skin (AOS) complex on stinging test score on women volunteers with hypersensitivity of the skin. The score was noted after 30 s, 5 min and 15 min of exposition with (a) 50% complex solution (T), (b) 100% complex solution and compared with nontreated skin (NT) after lactic acid application at 10%. The results were expressed by reporting the self-evaluation score on a scale of 0 to 3, where 3 is the maximal sting experienced. Graphs represent the mean (SEM) of each individual score for each volunteer (Wilcoxon test, ****P < 0.0001; *N* = 22). Evaluation of AOS complex on pruritus on pooled data from 33 participants aged 6 months to 3 years and from 33 participants aged 3 to 17 years declared by their parents as having atopic skin. Seven participants were excluded from the study. The complex was applied every day for 21 days, followed by 2 days without application. Pruritus score (c) as noted on day (D) 0, D21 and D23. Results are expressed by reporting the score from dermatologist on a scale of 0 to 10, where 10 is the maximal score. Graphs represent the mean (SEM) of each individual score for each participant (Wilcoxon test, *P < 0.05; ***P < 0.001; ****P < 0.0001; *N* = 59). mn, minute; ns, not significant.

To evaluate the effect of the AOS complex on the unpleasant sensations linked to pruritus, a stinging test was performed on women volunteers and a questionnaire was given to the parents of child volunteers with AD. The AOS complex in solution at 50% and 100% induced a significant decrease in the unpleasant sensation in the stinging test after 30 s [50% NT: 2.09 (0.16) vs. T: 0.91 (0.15); 100% NT: 2.36 (0.12) vs. T: 1.36 (0.15)], 5 min [50% NT: 1.55 (0.18) vs. T: 0.41 (0.13); 100% NT: 2.46 (0.14) vs. T: 0.59 (0.13)] and 15 min [50% NT: 0.68 (0.18) vs. T: 0.09 (0.09); 100% NT: 2.23 (0.17) vs. T: 0.45 (0.16)] of exposure (Figure 3a, 3b). Pruritus was also significantly decreased with mean (SEM) a score of 4.8 (0.2) at D0 vs. 1.2 (0.2) at D21, and 1 (0.2) at D23 for the balm (Figure 3c).

**Figure 4:**
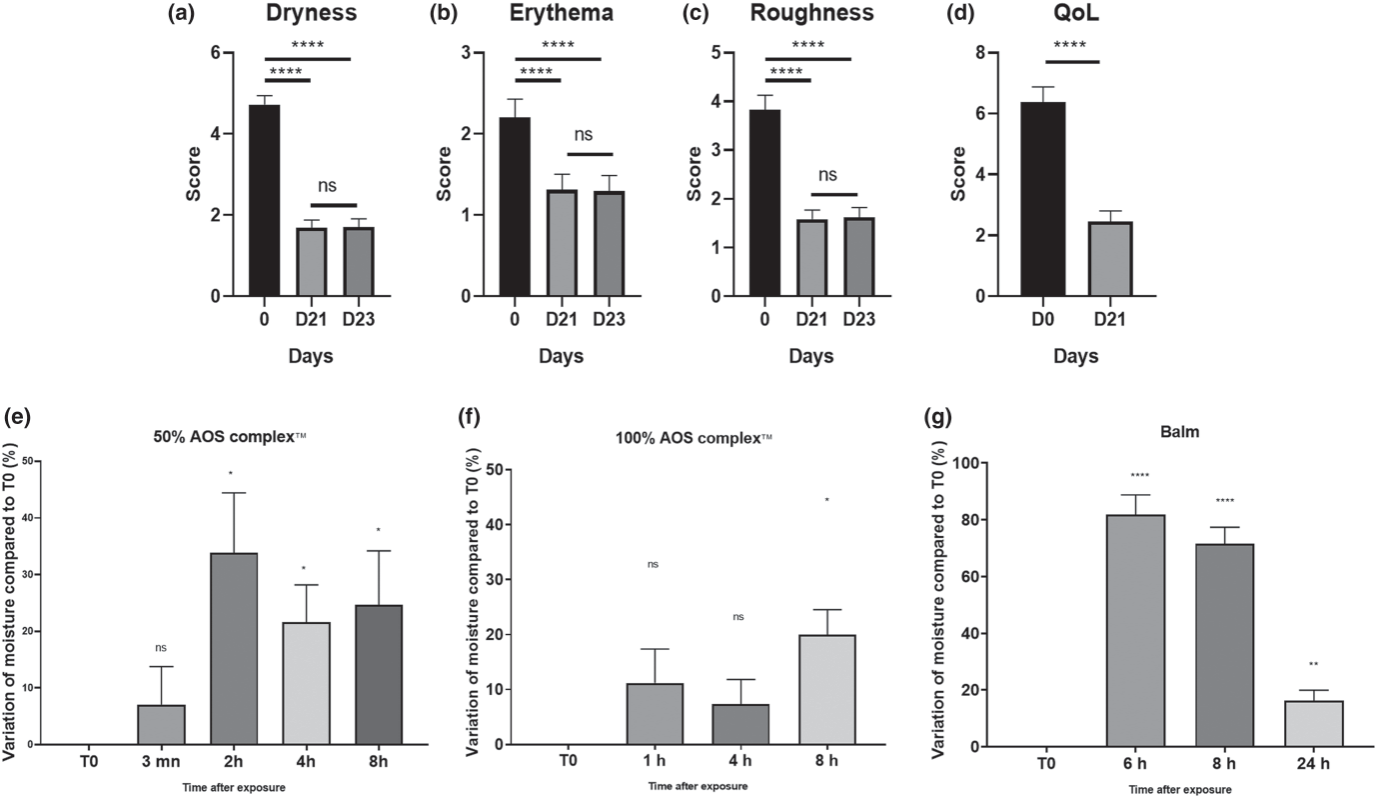
Evaluation of Active Oligo Skin (AOS) complex™ on dryness, erythema, roughness and quality of life (QoL) on pooled data from 33 participants aged 6 months to 3 years and 33 particpants aged 3 to 17 years declared by their parents as having atopic skin. Seven participants were excluded from the study. The complex was applied every day for 21 days, followed by 2 days without application. Scores for (a) dryness, (b) erythema, (c) roughness and (d) QoL were assessed with the help of the parents, with a maximum score of 45. Graphs represent the mean (SEM) of each individual score for each participant (Wilcoxon test, *P < 0.05; **P < 0.01; ****P < 0.0001; *N* = 59). Effect of AOS complex™ on skin moisture was evaluated by corneometry on women participants. The results were expressed as percentage of variation compared with the beginning of the measurement (T0) and nontreated zone (e) at 3 min, 2 h, 4 and 8 h after exposure to the complex at 50%, (f) at 1 h, 4 h and 8 h after exposure to the complex at 100%, or (g) at 6 h, 8 h and 24 h with balm. Graphs represent the mean (SEM) of measure score for each participant (one-way ANOVA post-hoc Dunnett’s, *P < 0.05; **P < 0.01; ****P < 0.0001; *N* = 11). ns, not significant.

With regard to quality of life, four parameters, dryness, erythema and roughness of atopic skin on child volunteers were evaluated. The balm induced a significant mean (SEM) decrease in dryness after daily application for 21 days followed by 2 days without [4.7 (0.2) at D0 vs. 1.7 (0.2) at D21 and 1.7 (0.2) at D23] (Figure 4a). The balm also induced a significant mean (SEM) decrease in erythema [2.2 (0.2) at D0 vs. 1.3 (0.2) at D21 and 1.3 (0.2) at D23] and roughness [3.8 (0.3) at D0 vs. 1.6 (0.2) at D21 and 1.6 (0.2) at D23] (Figure 4b, 4c). Quality of life was significantly increased, with [6.4 (0.5) at D0 vs. 2.5 (0.3) at D21 on the Children’s Dermatology Quality of Life Index/Infants’ Dermatology Quality of Life Index] (Figure 4d; for detailed quality of life data, see Figure S1 in Supporting Information). Regarding the moisturization assays performed with a Corneometer^®^ on women volunteers, the moisture of the skin was not significantly increased at 3 min but was significantly increased by a mean (SEM) of 33.9% (10.5), 21.6% (6.6) and 24.7% (9.5) at 2, 4 and 8 h, respectively, after exposition with AOS ­complex solution at 50% (Figure 4e), compared with the NT zone at the same time. Skin moisture was not significantly increased after 1 h and 4 h of exposure, but was significantly increased by a mean (SEM) of 19.96% (4.56) after 8 h after exposition to the AOS complex solution 100% (Figure 4f). The moisturizing balm induced a significant increase by a mean (SEM) of 81.8% (7), 71.5% (5.8) and 16.3% (3.7) of moisture after 6, 8 and 24 h of exposure (Figure 4g).

## Discussion

The objective of this work was to evaluate the *in vitro* and *in vivo* performance of the AOS complex on atopic skin. *In vitro*, the complex modulated bacterial biofilm formation and growth, decreased cytokine (IL-1, IL-6, IL-4) and neuropeptide (SP) but not TNF-α release, and increased the expression of ZO-1, CL1 and CL4. On volunteers with dry skin, the complex and the balm had a moisturizing effect after 1 h of application. Dryness and roughness were also reduced in young participants with AD. The balm decreased erythema and pruritus after 21 days of topical application by 60 young participants. On 22 adults, the stinging score was decreased after application of the AOS complex.

The AOS complex had no effect or a similar effect on the microbiota to that of PW on S. *epidermidis* growth and adhesion. Moreover, monospecies biofilms of *S. aureus* and *S. epidermidis* were affected by the AOS complex, with a larger effect on mixed biofilms. In summary, the AOS complex seems to strenghten *S. epidermidis* competition against *S. aureus*. The part of the biofilm occupied by *S. aureus* was reduced more than that occupied by *S. epidermidis*, indicating that the complex tends to favour the installation of the commensal bacterium *S. epidermidis*, although the differences between the two bacteria remain limited, suggesting that the risk of long-term dysbiosis is limited. An antagonism between *S. epidermidi*s and *S. aureus* has been demonstrated, particularly in biofilm formation.^46,47^

Topical calcineurin inhibitors (TCIs) show good anti-inflammatory and very good antipruritic effects in AD, while lacking some adverse effects associated with TCIs such as skin atrophy. TCIs are especially useful in patients who require long-term treatment.^20^ Two TCIs, tacrolimus ointment and pimecrolimus cream, are licensed for topical AD treatment. The anti-inflammatory effects result from an inhibition of proinflammatory cytokine(s) production by T cells and mast cells, and a reduction of SP release in culture keratinocyte and neuron co-cultures,^37,48^ whereas a part of their antipruritic effects can be attributed to a specific effect on TRPV1+ skin neurons.^20,49^ In our study, we showed a potent anti-TRPV1, or this resulting signalling pathway, effect in capsaicin (TRPV1 agonist)-induced inflammation in co-culture models. Furthermore, the AOS complex inhibits the release of cytokine IL-4, and SP but not of TNF-α (no more than placebo gel). This is consistent with a reduction of erythema, stinging sensation and pruritus. SP release is a quick phenomenon (15 min), suggesting a rapid effect of the complex. This result is in accordance with the results of the *in vivo* stinging tests. Interleukins are more long-term inflammation-regulatory factors and variations in their expression could explain the anti-inflammatory effect of the AOS complex observed in children. Concerning IL-4 release, the 50% AOS complex-containing gel had the same effect as the balm, but, surprisingly, no effect was noted when the concentration was increased to 100%, suggesting that an optimal concentration of minerals is needed. It is possible that interactions occur between ions at high concentrations leading to the formation of nonsoluble and inactive forms. Interestingly, placebo composed of a gel alone without complex possessed an intrinsic effect, and decreased the release of IL-1, IL-6 and TNF-α induced by PMA.

The AOS complex formulated in the balm induced a reduction in roughness and dryness, and increased the skin moisturization. The balm induced an amplified effect compared with AOS alone, due to an inherent effect of balm composition. Intercellular junction proteins, although not the only element involved, are implicated in the barrier function and preservation of the skin’s physiological integrity by controlling water loss. We showed that after 48 h application, the AOS complex induced an increase in CL1 and CL4 expression. This effect was higher in balm than in solution, presumably because of other ingredients in the balm such as emollients, which could increase AOS complex penetration. The placebo balm contains all ingredients except the mineral solution. A synergistic effect of the composition of balm and complex is also possible. This increase can potentially explain the *in vivo* observations. It is interesting to note that observations of these junction proteins 24 h, 72 h or 7 days after balm application (data not shown) did not show noticeable modification of expression, suggesting that a delay and regular applications are probably required for efficacy of the AOS complex.

The AOS complex alone or formulated in balm appears to display *in vitro* and *in vivo* potent antipruritic and anti-­inflammatory activities in the AD context, suggesting a benefit of its use in baseline AD management as recommended task forces.^17–20^ The effect of the complex is probably partly related to its unique mineral composition, including calcium, potassium and high concentrations of magnesium and sodium.

New clinical trials are necessary to confirm these results, such as double-blinded vs. balm placebo (placebo already displays active ingredients). It is important to decipher the exact mechanism of action of the AOS complex by other complementary and more robust *in vitro* techniques to confirm and challenge these initial interesting data.

## Supplementary Material

vzae029_Supplementary_Data

## Data Availability

The data underlying this article will be shared on reasonable request to the corresponding author.
