# Supplementary material for: In vitro and in vivo efficacy of the Active Oligo Skin complex™, a new active ingredient processed from seawater, on multiple parameters of atopic skin
Source: Skin Health Dis. 2025 Feb 14;5(1):22–30. doi: 10.1093/skinhd/vzae029 (PMC11924379; doi:10.1093/skinhd/vzae029)
Supplement: vzae029_Supplementary_Data [file vzae029_supplementary_data.docx]

Supplementary Data

Ethical statements

The research complies with ethical principles and applicable international, EU, and French law, in particular, EU directives 2004/23/EC and 2010/63/EU. For *in vitro* experiments, human skin explants were obtained from abdominal reduction surgery after the donor gave their written informed consent (DC-2014-2319). The study was approved by the local ethics committee and realized in accordance with European Law. *In vivo* in-use tests were performed, under dermatological control, in accordance with European Law, all participants were volunteers and gave written informed consent. Animal samples were realized in accordance with 3R principle and EU principle.

**Methods**

**Bacterial growth and biofilm formation**

1. Bacterial strains and culture conditions

*S. epidermidis* MFP04 was isolated from the skin of healthy volunteers and characterized by complete genome sequencing. It was grown at 37°C in aerobic condition in Brain-Heart-Infusion medium (BHI) with 2% glucose. NR-51164 is a *Staphylococcus aureus* strain RN4220 containing the DsRed.T3(DNT) red fluorescent protein (mCherry) reporter plasmid pSRFPS1. It was obtained from the American strain library BEI Resources (www.beiresources.org). This strain was grown at 37°C in aerobic condition and under agitation (180 rpm/mn) in Tryptic Soy Broth (TSB) with trimethoprim 10 µg/mL.

*2. Bacterial growth kinetics*

For monitoring of *S. epidermidis* growth, an overnight bacterial pre-culture in liquid medium was diluted at OD_580nm_ = 0.08 in fresh medium and 200 µL and dropped in sterile 100-wells flat-bottomed plastic culture plates. The plates were incubated for 24 h at 37°C under constant agitation. The OD of each well was measured at 580 nm every 30 min. All studies were carried out in quadruplicate.

*4. Study of biofilm formation by confocal laser scanning microscopy*

Biofilms were produced in thin flat glass 24-wells plates. At the end of the incubation period, wells were washed with sterile PW and stained with Syto 9 Green Fluorescent Nucleic Acid Stain 1µM for observation of *S. epidermidis* MFP04. For observation of the mCherry transformed *S. aureus* strain no straining was necessary. All biofilms were fixed with ProLong Diamond Antifade and observed using a Zeiss LSM710 confocal laser scanning microscope. Syto 9 and mCherry fluorescence emission were detected at 498 and 610 nm, respectively. For visualization and processing of three-dimension images, Zen 2.1 SP1 software was used. Quantitative analyses of images stacks were performed using COMSTAT2 software. Biomass volume (μm^3^/μm^2^), maximal and average thickness (μm) were determined using ImageJ software.

Effect of the Active Oligo Skin complex on Interleukin 4 release induced by lactic acid on reinnervated skin explants

Dorsal root ganglia (DRG) cells were extracted from 3-4 days old wistar rats after appropriate sacrifice. After enzymatic and mechanical dissociation of DRG, isolated cells were seeded in quadruplicate for each condition in 96 well plates in medium containing DMEM/HAM F12 (3/1), B27 (50x), insulin (5 µg/mL), hydrocortisone (10 ng/mL), nerve growth factor NGF (25 ng/mL), and brain derived neurotrophic factor BDNF (10 ng/mL). The same day, skin explants were placed in wells at the air-liquid interface and grown in this position for 9 days constituting a reinnervated skin model. Balm, placebo or the gel with or without AOS complex^TM^ were deposited at the surface of skin explants. Five minutes later, lactic acid (10% in PBS) was applicated. After 24h of culture, supernatants were collected and IL4 was quantified using a Duoset kit according to the manufacturer’s instructions (R&D).

Effect of the Active Oligo Skin complex^TM^ on Interleukin 1, 6 and TNFα release induced by phorbol myristate acetate (PMA) in skin explants

Skin explants were placed in DMEM during 4h and subsequently pre-treated for 24h with gels containing the AOS complex^TM^ at 0%, 2%, 10 or 50% or with dexométhaxone (100 µM in the culture medium) as positive control. Then, PMA (1µg/mL) was applied in the medium of explants previously treated or not by the formula. After 24h of incubation at 37°C, supernatants were collected to quantify IL6, IL1 and TNFα by ELISA kit.

Effect of the Active Oligo Skin complex^TM^ on capsaicin induced Substance P (SP) release by keratinocytes and neurones.

After 3 days of culture of DRG cells, the medium was removed, and human primary keratinocytes were added in culture for one supplementary day in KSFM®. For assessment of SP release, the medium was replaced by for 15 min with fresh medium containing capsaicin 10 µM alone dissolved in dimethysulfoxide (DMSO 0.02%), medium with capsaicin and AOS complex^TM^ 50% or 100%, or capsaicin solvent (DMSO 0.02%) alone. Physiological serum (PW, NaCl 0.9%) was used as a control of the AOS complex^TM^. Supernatants were collected, mixed with antiproteases and SP was quantified using a Cayman kit (583751) according to the manufacturer’s instructions.

Effect of the Active Oligo Skin complex^TM^ on Zonula Occludens 1 and Claudine 1 and 4 expression in skin explants

Human skin explants were incubated at the air-liquid interface during 7 days in DMEM/F12. At 5 days, the AOS complex^TM^ (50 or 100 %) mixed in 4% methyl cellulose gel, placebo (gel not supplemented with AOS complex), balm and placebo balm were applied with small spatula on the epidermis surface. After 48h of incubation, skin explants were placed in RNAlater. Tissues were disrupted using Precellys and ARN were extracted using RNEASy mini kit (Qiagen). Quantity and quality of RNAs were checked by analysis of the 260/280 nm adsorption ratio. After reverse transcription, qPCR was realized using Power Sybrgreen in thermocycler (Stepone, Applied Biosystem). Gene expression modulation was evaluated using 2^(-ddct) method with actin as housekeeping gene. Probes designed for the study are presented in Supplementary Table 1.

*In-vivo* evaluation of the effect of the Active Oligo Skin complexTM on skin moisturizing

The moisturizing potential of the AOS complex^TM^ was determined by repeated measure of the skin electrical capacity on treated (T) and non-treated (NT) control sites. Measurements were realized using a corneometer® at the onset of the experiment (T0) and after 1, 4 and 8 hours (T1h, T4h and T8h) on 11 female adult volunteers (average age 61 years) with legs dry skin. The AOS complex^TM^ 100% was tested after 3mn, 2h, 4h and 8h application on 10 volunteers and at 0h, 6h, 8h and 24h for the 50% Balm.

*In-vivo* evaluation of the effect of the Active Oligo Skin complexTM on the stinging sensation

The stinging sensation was evaluated using a classical methodology using a randomized site on 22 female adult volunteers (average age 44 years) for the product containing 50% AOS complex^TM^ and on 22 female adult volunteers (average age 38 years) for the product with 100% AOS complex^TM^. Briefly, after application of 10% lactic acid on both nasolabial folds, an evaluation of the stinging sensations perceived by the subjects on each site was realized at 30s, 5min and 15min. Hence, the studied product was applied on the treated site (T) of the determined nasolabial fold according to the randomization list, the other side remaining untreated (NT).

*In-vivo* evaluation of the effect of the Active Oligo SkinTM complex on atopic dermatitis symptoms and quality of life of young patients

The quality of life, skin aspect (Dryness, erythema, roughness) and pruritus sensation was evaluated under dermatological control in 33 subjects from 6 months to 3 years and in 33 subjects from 3 to 17 years with a pruritus score (scratching sensation) ≥ 3 on a scale from 0 to 10 and with atopic skin (not in crisis) (symptoms based on parents’ declarations). The product was applied each day for 21 days (D21) and the study was completed with 2 supplementary days without application of the product (D23).

Supplementary figure 1

A

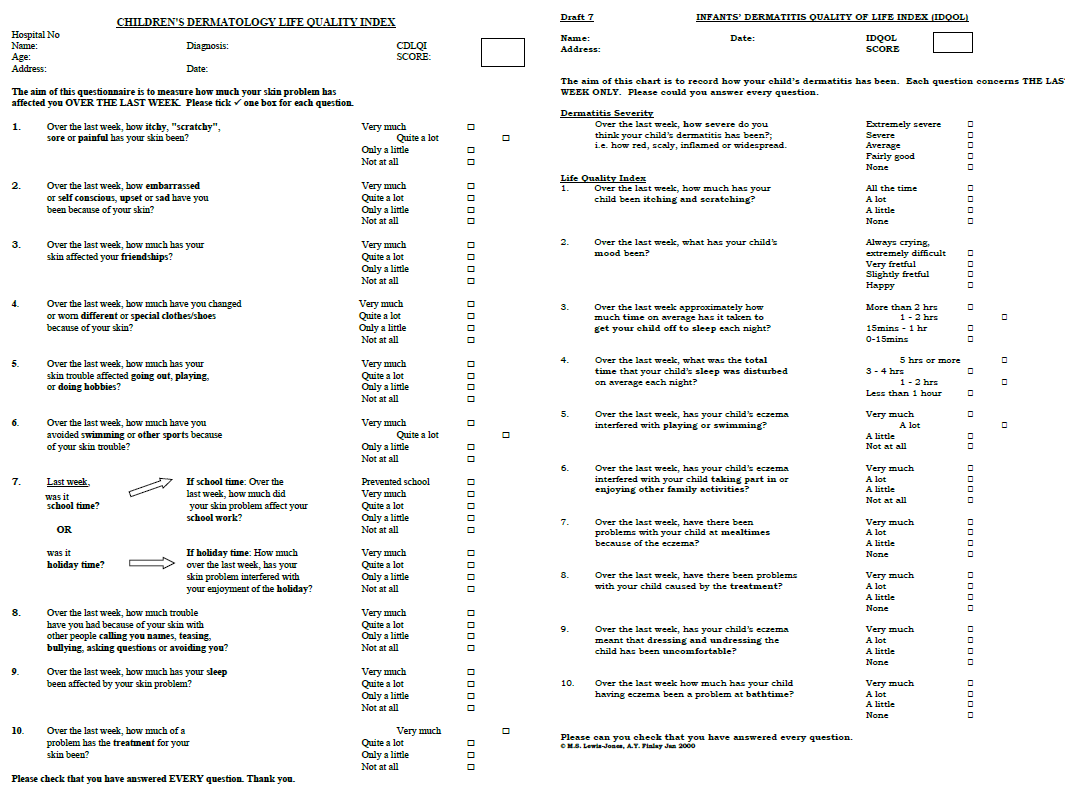


B

**Figure S1 Evaluation of Active Oligo Skin complex^TM^ on QoL on pooled 33 subjects from 6 months to 3 years old and 33 subjects from 3 to 17 years old declared by parents as atopic skin.** 7 subjects were excluded from the study. The complex was applied every day during 21 days following by two days without application**.** QoL was assessed with help of the parents. A) Graph represent the mean of each individual score for each subjects for each question at D0 and D21 +/- SEM. B) Questionary

Supplementary table 1: probe used for quantitative PCR

| Probe | Forward | Reverse |
| --- | --- | --- |
| Zonula Occludens 1 | CCT-TCG-GGG-ACC-ATC-AAG-AC | GCC-TGC-CCA-CTT-CTT-CAG-TT |
| Claudine 1 | TCG-ACC-AAT-GCT-CTC-TCA-GC | CTC-CTG-GAG-GAG-AGG-TCC-AT |
| Claudine 4 | CCA-CTC-GGA-CTT-CCC-AA | ACT-TCC-GTC-CCT-CCC-CAA-TA |
| Actin | GAG-ACC-TTC-AAC-ACC-CCA-GC | ATG-TCA-CGC-ACG-ATT-TCC-CT |

Reference

1. O’Toole GA. Microtiter dish biofilm formation assay. *J Vis Exp JoVE*. 2011;(47):2437. doi:10.3791/2437
